# LUBAC determines chemotherapy resistance in squamous cell lung cancer

**DOI:** 10.1084/jem.20180742

**Published:** 2019-02-04

**Authors:** E. Josue Ruiz, Markus E. Diefenbacher, Jessica K. Nelson, Rocio Sancho, Fabio Pucci, Atanu Chakraborty, Paula Moreno, Alessandro Annibaldi, Gianmaria Liccardi, Vesela Encheva, Richard Mitter, Mathias Rosenfeldt, Ambrosius P. Snijders, Pascal Meier, Marco A. Calzado, Axel Behrens

**Affiliations:** 1 Adult Stem Cell Laboratory, The Francis Crick Institute, London, UK; 2 Instituto Maimónides de Investigación Biomédica de Córdoba, Córdoba, Spain; 3 Unidad de Cirugía Torácica y Trasplante Pulmonar, Hospital Universitario Reina Sofía, Córdoba, Spain; 4 Breast Cancer Now, Toby Robins Research Centre, Institute of Cancer Research, London, UK; 5 Proteomics, The Francis Crick Institute, London, UK; 6 Bioinformatics and Biostatistics, The Francis Crick Institute, London, UK; 7 Comprehensive Cancer Center Mainfranken, University of Würzburg, Würzburg, Germany; 8 Departamento de Biología Celular, Fisiología e Inmunología, Universidad de Córdoba, Córdoba, Spain; 9 Faculty of Life Sciences and Medicine, King’s College London, London, UK

## Abstract

Lung squamous cell carcinoma (LSCC) and adenocarcinoma (LADC) are the most common lung cancer subtypes. Molecular targeted treatments have improved LADC patient survival but are largely ineffective in LSCC. The tumor suppressor FBW7 is commonly mutated or down-regulated in human LSCC, and oncogenic *KRasG12D* activation combined with *Fbxw7* inactivation in mice (KF model) caused both LSCC and LADC. Lineage-tracing experiments showed that CC10^+^, but not basal, cells are the cells of origin of LSCC in KF mice. KF LSCC tumors recapitulated human LSCC resistance to cisplatin-based chemotherapy, and we identified LUBAC-mediated NF-κB signaling as a determinant of chemotherapy resistance in human and mouse. Inhibition of NF-κB activation using TAK1 or LUBAC inhibitors resensitized LSCC tumors to cisplatin, suggesting a future avenue for LSCC patient treatment.

## Abstract (precis)

This study identifies a novel therapeutic strategy against cisplatin-resistant lung squamous cell carcinoma (LSCC) using mouse models and patient samples. LSCC chemoresistance depends on LUBAC and high NF-κB activity, mechanisms that can be targeted to increase therapy response.

## Introduction

Lung cancer is the most common epithelial tumor and the leading cause of cancer death worldwide. It is histologically differentiated into small cell lung cancer (SCLC) and non–small cell lung cancer (NSCLC). NSCLC tumors can be further subdivided into lung adenocarcinoma (LADC), squamous cell carcinoma (LSCC), and the rarer large cell carcinoma. Progress has been made in the targeted treatment of LADC, largely due to the development of small-molecule inhibitors against epidermal growth factor receptor (EGFR), anaplastic lymphoma receptor tyrosine kinase (ALK), and ROS1 (Cardarella and Johnson, 2013). However, such treatments have proved ineffective for LSCC patients (Novello et al., 2014; Hirsch et al., 2017). This, together with the lack of LSCC-specific therapeutic targets, has resulted in few recent significant advances in the treatment of this disease (Liao et al., 2012; Gandara et al., 2015). Consequently, despite its limited effectiveness on disease progression and prognosis, platinum-based chemotherapy remains the cornerstone of current treatment for LSCC (Scagliotti et al., 2008; Fennell et al., 2016; Isaka et al., 2017). Therefore, elucidating the critical molecular pathways involved in LSCC is crucial to identify new therapeutic approaches.

Comprehensive genetic analyses of human LSCC samples revealed numerous genomic alterations in genes such as *PIK3CA*, *TP53*, *CDKN2A*, and *FBXW7* (Kan et al., 2010; Cancer Genome Atlas Research Network, 2012). The *FBXW7* protein product F-box/WD repeat-containing protein 7 (FBW7) is the substrate recognition component of a Skp, Cullin, F-box–type ubiquitin ligase, which targets several well-known oncoproteins, including c-Myc, Notch, and c-Jun, for degradation (Davis et al., 2014).

The NF-κB pathway is involved in multiple steps in tumorigenesis and chemoresistance (Zhang et al., 2017). In physiological conditions, this pathway is tightly regulated by ubiquitylation. Ubiquitin (Ub) chains regulate the degradation of the IκB proteins and also serve as a scaffolding, recruitment, and activation platform in receptor signaling complexes. Lysine-63 (K63)– and methionine-1 (M1)–linked ubiquitin chains mediate the key upstream events of recruiting TAK1 and the IKK complex, respectively, resulting in the activation of the NF-κB pathway (Jiang and Chen, 2011; Emmerich et al., 2013). The linear Ub chain assembly complex (LUBAC) specifically assembles M1-linked Ub chains on the IKK complex subunit NEMO/IKKγ. Recent findings suggest a role of LUBAC in tumor formation in which excessive LUBAC activation causes abnormal NF-κB signaling and tumor growth (Yang et al., 2014) and attenuates chemotoxicity in cell lines (MacKay et al., 2014). Although NF-κB activation has been reported in several tumors including lung cancer (Karin and Greten, 2005), the potential role of the LUBAC–NF-κB pathway in LSCC tumors is unknown.

Here, we describe a novel genetic mouse model in which the loss of *Fbxw7* concomitant with *KRasG12D* activation (KF mice) promoted the formation of two NSCLC cancers, LSCC as well as LADC. LADC and LSCC were found in distinct anatomical locations, as observed in humans. Whereas LADC exclusively formed in the alveolar space, LSCC was found near the airways. Club CC10^+^ cells, but not basal cytokeratin 5–positive (CK5^+^) cells, were the cells of origin of LSCC in the KF model. Moreover, we found that LSCC tumors were resistant to cisplatin chemotherapy and identified the LUBAC complex as a determinant of chemotherapy resistance. Inhibition of LUBAC or NF-κB signaling resulted in sensitization of LSCC tumors to cisplatin, suggesting a future avenue for LSCC patient treatment.

## Results

### FBW7 is frequently lost in human LSCC

**Figure 1.**
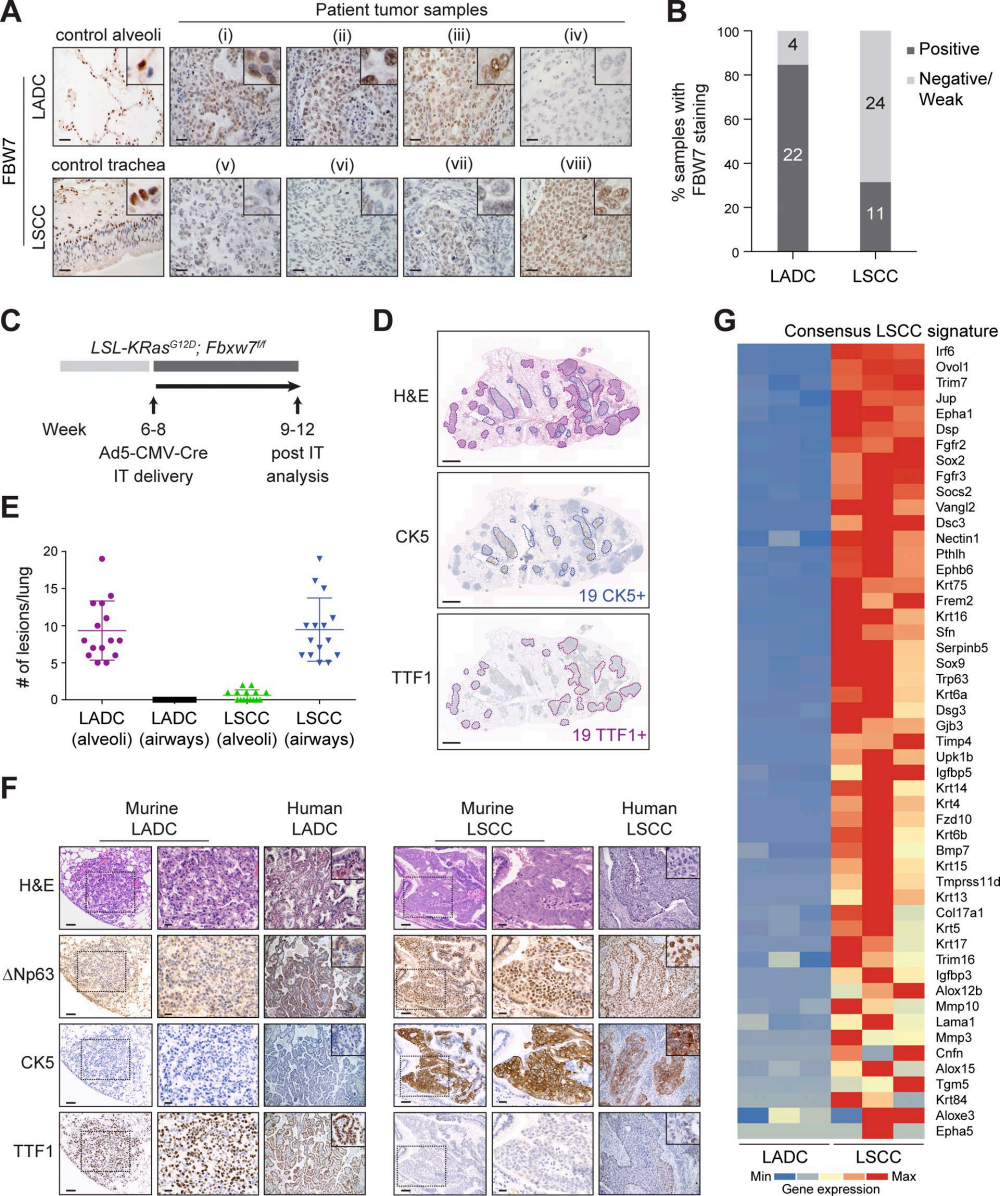
**Biallelic inactivation of *Fbxw7* and *KRas^G12D^* activation in the adult mouse lung leads to LSCC and LADC formation. (A)** Representative human lung LADC (i–iv) and LSCC (v–viii) tumors and control lung sections stained with FBW7 antibodies. Bars, 20 µm. **(B)** Quantification of FBW7 protein staining in human LADC and LSCC tumors as in A. *n* = 26 LADC, 35 LSCC. **(C)** Biallelic inactivation of *Fbxw7* and *KRas^G12D^* activation by intratracheal (IT) delivery of Ad5-CMV-Cre virus in the adult mouse lung as a model of NSCLC. **(D)** KF model develops LSCC (CK5^+^) and LADC (TTF1^+^) tumors. Sections representative of six animals. **(E)** Quantification and localization of mouse lung LADC and LSCC tumors in the KF model. *n* = 15 lungs. Plots indicate mean ± SD. **(F)** Human and mouse NSCLC samples were stained with biomarkers used clinically to distinguish LADC (TTF1) from LSCC (CK5 and ΔNp63) tumors. Bars, 100 µm (columns 1 and 4); 20 µm (columns 2 and 5). Sections representative of six animals. **(G)** Heat map of RNASeq data showing relative expression of LSCC genes in LADC and LSCC tumors from *n* = 3 mice of the KF genotype. Gene set shown is selected from gene sets up-regulated in *Lkb1^f/f^; Pten^f/f^* LSCC (Xu et al., 2014) and in *LSL-Sox2; Pten^f/f^; Cdkn2ab^f/f^* LSCC and human LSCC (Ferone et al., 2016). Genes are ordered according to z-score. See also Fig. S1.

Genomic studies of human LSCC have reported recurrent mutations in the *FBXW7* tumor suppressor gene (Kan et al., 2010; Campbell et al., 2016). Data from The Cancer Genome Atlas (TCGA) show 6.4% of human LSCC cases with mutations in *FBXW7*, the majority associated with loss of function (Fig. S1 A; Cerami et al., 2012). Moreover, immunohistochemistry (IHC) staining on human lung biopsy samples showed that only 31% (11/35) of LSCC tumor samples expressed detectable levels of FBW7 protein. In contrast, 85% (22/26) of human LADC samples were positive for FBW7 staining, and adjacent normal lung tissue was positive for FBW7 in all patients (Fig. 1, A and B). These data suggest that FBW7 is lost or down-regulated frequently in human LSCC, and hence that FBW7 inactivation could be a driver of human LSCC.

### *Fbxw7* inactivation, together with oncogenic *KRas* activation, results in LSCC as well as LADC tumors in the mouse lung

Conditional activation of oncogenic *KRas^G12D^* in the mouse lung is a well-established mouse model of human LADC (Jackson et al., 2001). *KRAS* mutations are very frequently observed in human LADC, but the RAS tumor driver pathway is also activated in up to half of human LSCC tumors, most commonly due to transcriptional up-regulation and amplification of *KRAS* and the upstream receptor tyrosine kinases *EGFR* and *FGFR1* (Fig. S1 A; Cancer Genome Atlas Research Network, 2012; Campbell et al., 2016).

Intratracheal administration of Cre-expressing adenovirus in *LSL-KRas^G12D^* mice results in the formation of LADC (DuPage et al., 2009). To investigate whether genetic loss of *Fbxw7* concomitant with oncogenic *KRas^G12D^* activation would alter the lung tumor type produced in this model, we generated a conditional *LSL-KRas^G12D^*; *Fbxw7^f/f^* (KF) mouse strain. Animals were administered with adenovirus expressing Cre recombinase under the control of the ubiquitous CMV promoter (Ad5-CMV-Cre) via intratracheal intubation, and the lungs were analyzed 9–12 wk after virus administration (Fig. 1 C).

Control *LSL-KRas^G12D^* mice developed only LADC, as previously reported (Jackson et al., 2001; Fig. S1 B). However, 100% of KF mice developed two distinct types of NSCLC upon Cre-mediated recombination: a tumor type that resembled LADC, and a second lung cancer subtype resembling LSCC (Fig. 1 D). The ratio of LSCC to LADC lung tumors formed in the KF mouse model was on average 1:1 (Fig. 1 E).

The two tumor types were blindly analyzed and classified by a clinical pathologist as LADC and LSCC according to the most recent World Health Organization criteria (Travis et al., 2015). Both tumor types resembled their human counterparts histologically (Fig. 1 F). LADC occurred exclusively in alveolar lung tissue and displayed solid, acinar, lepidic, and papillary growth patterns. Moreover, LADC cells were positive for TTF1 and negative for CK5 and ΔNp63 staining (Fig. 1 F). LSCC occurred mainly in bronchi (occasionally manifesting as small foci in alveolar lung tissue), showed largely solid growth patterns, with occasional intracellular bridges and rare keratinization, and were negative for TTF1 but expressed CK5 and ΔNp63 (Fig. 1 F). We observed infiltration of neutrophils, but not macrophages, CD4^+^, or CD8^+^ T cells. As has been described for the *Lkb1^f/f^*, *Pten^f/f^* LSCC model, the immune evasion markers PD-1 and PD-L1 were expressed in KF LSCC tumors, and they were positive for NGFR and Sox2, two other markers shown to be characteristic of human and mouse LSCC (Fig. S1 C; Xu et al., 2014).

To further characterize the tumor types arising in the KF model, we isolated LADC and LSCC cells from KF lungs and performed transcriptome analysis (RNASeq). LSCC cells displayed a strong LSCC gene signature, selected from published gene sets up-regulated in *Lkb1^f/f^*, *Pten^f/f^* LSCC (Xu et al., 2014), *LSL-Sox2; Pten^f/f^; Cdkn2ab^f/f^* LSCC, and human LSCC (Ferone et al., 2016; Fig. 1 G). LADC cells from the same animals did not express this gene signature, underlining that distinct tumor types arise from the same oncogene combination in the KF model (Fig. 1 G). One possibility that could explain this duality is that LSCC and LADC tumors arise from different cells of origin. We therefore investigated the cell of origin of LSCC in the KF model.

### Basal cells do not give rise to LSCC in the KF model

Different cell types have been reported to give rise to LSCC (Ferone et al., 2016). Basal cells are considered potential cells of origin of LSCC, as they share certain markers with LSCC tumor cells, including cytokeratin 5 expression. KF animals were therefore crossed with two Cre lines driving recombination in basal cells, using cytokeratin 5 or cytokeratin 14 promoters (*CK5-Cre^ERT^* or *CK14-Cre^ERT^* mice; Indra et al., 1999; Vasioukhin et al., 1999). Both *CK5-Cre^ERT^*; KF and *CK14-Cre^ERT^*; KF mice developed skin and life-limiting oral tumors after 3–4 wk. At that time no lung tumors were detectable (data not shown), so the analysis of lung tumorigenesis at later stages was precluded.

To be able to analyze LSCC development at later time points, we generated recombinant viruses expressing Cre recombinase driven by the cytokeratin 5 promoter (Ramirez et al., 2004). To validate the specificity of Ad5-CK5-Cre virus, Rosa-CAG-LSL-tdTomato lineage tracer mice were treated with naphthalene to deplete Club secretory cells and to expose the basal cell layer, and high-titer recombinant viruses were then administered intratracheally. Targeted cells were identified by IHC staining for RFP/Tomato and cellular location. At 3 and 12 wk following Ad5-CK5-Cre infection, traced cells were found lining the basal layer of tracheal sections and showed CK5-positivity (Fig. S1 D), validating the basal cell targeting of the virus. However, KF animals treated with naphthalene and Ad5-CK5-Cre did not develop lung tumors even after a long latency (30 wk; Fig. S1 E). Thus, targeting *Fbxw7^Δ/Δ^* and *KRas^G12D^* specifically to CK5-expressing cells failed to give rise to LSCC.

### CK19^+^ luminal cells give rise to LSCC in the KF model

Because basal cells appear not to be the cell of origin of KF LSCC, we next tested whether luminal epithelial cells function as cells of origin in the KF lung cancer model. KF mice harboring the *R26-LSL-YFP* lineage tracer were crossed to *CK19-Cre^ERT^* mice to target the oncogenes to CK19-expressing cells in the adult lung, including club, ciliated, and alveolar cells (Iyonaga et al., 1997; Cole et al., 2010).

*CK19-Cre^ERT^;* KF*; R26-LSL-YFP* (CKFY) mice developed both LSCC and LADC tumors in an ∼1:1 ratio (Fig. S2 A). The histopathology of both tumors in CKFY mice was identical to that observed following Ad5-CMV-Cre infection. LSCC tumors were located in and adjacent to the airways and expressed CK5 and ΔNp63, whereas LADCs were located in the alveoli and positive for TTF1 and Sftpc (Fig. S2 A). These data suggest that KF LSCC may initiate in luminal CK19^+^ cells that subsequently change their marker expression to give rise to the squamous phenotype.

### CC10^+^ luminal cells initiate LSCC in the KF model

**Figure 2.**
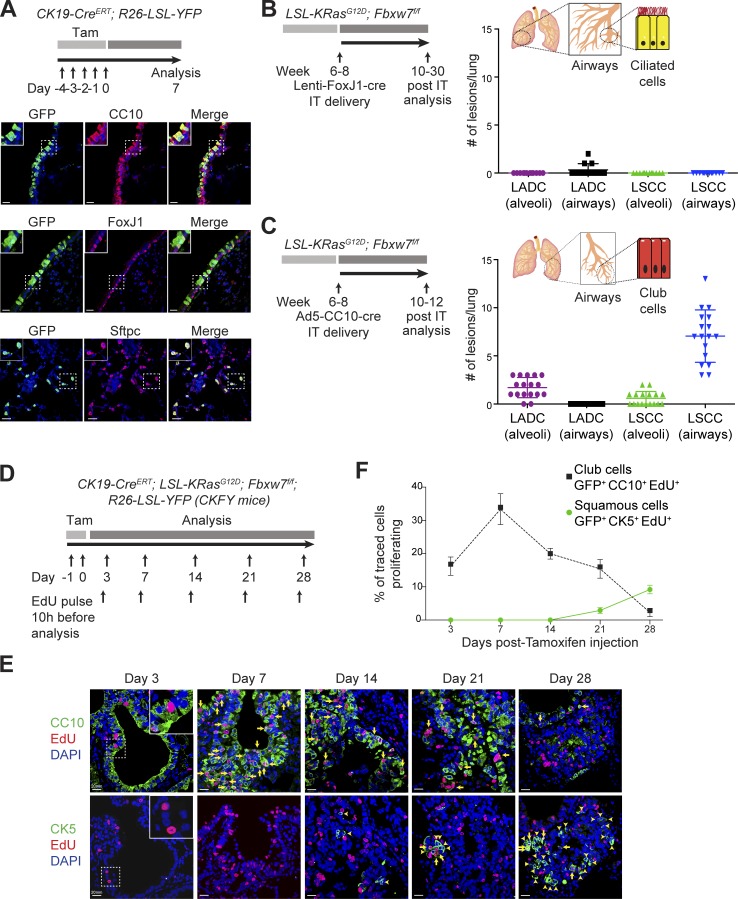
**Lung LSCC originates from CC10^+^ luminal cells in the KF model. (A)** Immunofluorescent staining for CC10, FoxJ1, Sftpc, and GFP in *CK19-Cre^ERT^; R26-LSL-YFP* mouse lung sections at 1 wk after induction. Images representative of three animals. Bars, 20 µm. Tam, tamoxifen. **(B)** Ciliated FoxJ1^+^ cells were targeted by intratracheal (IT) delivery of Lenti-FoxJ1-Cre virus to KF animals. The graph shows the quantification and localization of lung tumors produced. *n* = 12 lungs. Plots indicate mean ± SD. **(C)** Club CC10^+^ cells were targeted by intratracheal delivery of Ad5-CC10-Cre virus to KF animals. The graph shows the quantification and localization of lung tumors produced. *n* = 17 lungs. Plots indicate mean ± SD. **(D)** Scheme for analysis of cell proliferation in *CK19-Cre^ERT^; KRas^G12D^*; *Fbxw7^f/f^; R26-LSL-YFP* (CKFY) mice. **(E)** Lung serial sections from CKFY mice were immunostained for CC10, CK5, and EdU at 3, 7, 14, 21, and 28 d after tamoxifen injection. Arrows indicate proliferating cells. Arrowheads indicate double CK19^+^CK5^+^ cells. Images representative of five animals. Bars, 20 µm. **(F)** Quantification of proliferating CC10^+^ and CK5^+^ cells at the indicated time points. Graph indicates mean ± SD of five animals. See also Figs. S1, S2, and S3.

To identify the CK19^+^ luminal cell initiating LSCC tumors, we performed lineage tracing in the *CK19-Cre^ERT^; R26-LSL-YFP* mouse model. Lineage-labeled CK19 cells in the airways were positive for the club cell marker CC10 or the ciliated cell markers FoxJ1 and acetylated tubulin (AcTub) and negative for the goblet cell marker mucin 5Ac (Muc5Ac; Fig. 2 A and Fig. S2 B). Expression of FoxJ1 and CC10 did not overlap, enabling ciliated and club cells to be distinguished in the airways (Fig. S2 C). In the alveoli, CK19-traced cells were positive for Sftpc, a marker of alveolar type II cells (Fig. 2 A; Barkauskas et al., 2013).

To target these cell types individually, we used recombinant viruses expressing Cre recombinase under the control of the FoxJ1 promoter (Lenti-FoxJ1-Cre), the CC10 promoter (Ad5-CC10-Cre), or the Sftpc promoter (Ad5-Sftpc-Cre) to initiate tumorigenesis in ciliated, club, and alveolar type II cells, respectively (Zhang et al., 2007; Sutherland et al., 2011).

KF mice infected intratracheally with Lenti-FoxJ1-Cre virus did not develop LSCC tumors even after a long latency (30 wk; Fig. 2 B). Ciliated FoxJ1^+^ cells were able to give rise to very few lung tumors (average 0.3 per lung) located in and adjacent to the airways, but these were positive for TTF1 and Sftpc, not CK5 or ΔNp63, suggestive of LADC (Fig. 2 B and Fig. S3 A).

In contrast, KF mice infected with Ad5-CC10-Cre virus developed tumor lesions with histological characteristics of LSCC and were mainly located in and adjacent to the airways. Markers characteristic of human LSCC, CK5 and ΔNp63, were detected in these tumors (Fig. 2 C and Fig. S3 B). LSCC tumors were also observed in the alveoli, although at low frequency (Fig. 2 C). Ad5-CC10-Cre infection also resulted in LADC tumors, which were found exclusively in the alveolar space and displayed high expression of TTF1 and Sftpc as expected (Fig. 2 C and Fig. S3 B). CC10^+^ cells originated predominantly LSCC (80%), with the remainder of the lesions being LADC tumors (Fig. 2 C).

It has recently been reported that alveolar type II cells also have the ability to form LSCC (Ferone et al., 2016). However, targeting alveolar type II cells with Ad5-Sftpc-Cre in KF mice resulted exclusively in TTF1- and Sftpc-expressing adenomas and adenocarcinomas distributed in the alveolar area (Fig. S3 C). Thus, whereas LADC tumors in the KF mouse model originate from Sftpc^+^ alveolar cells, LSCC tumors initiate in CC10^+^ luminal cells of the airways.

To analyze the progression of early LSCC lesions in the airways, we performed lineage tracing in the CKFY mouse model in combination with ethynyl deoxyuridine (EdU) staining to track the proliferation of targeted cells (Fig. 2 D). To trace only a small number of cells, we used a low dose of tamoxifen to induce recombination. Lung sections were costained for EdU and CC10 or CK5 to monitor tumor initiation. 16% of traced club (GFP^+^CC10^+^) cells proliferated as early as 3 d after tamoxifen injection, as indicated by EdU incorporation (Fig. 2, E and F; and Fig. S3 D). At 7 d, we observed a thickening of the airway epithelium, and CC10^+^ cells continued to proliferate strongly, with a third of traced CC10^+^ cells incorporating EdU. We began to observe lineage-traced CK5^+^ cells in the airways only around day 14 after recombination, and their numbers subsequently increased, accompanied by a decrease in CC10^+^ proliferating cells (Fig. 2, E and F; and Fig. S3 D). At 28 d after tamoxifen injection, we found traced lesions resembling LSCC in the airways, which were CK5^+^ and negative for CC10 (Fig. 2, E and F; and Fig. S3 D). These data support the notion that LSCC initiates in luminal CK19^+^ CC10^+^ cells that subsequently transdifferentiate to give rise to the squamous phenotype.

### Primary KF LADC and LSCC tumor cells retain their identity and tumorigenic capacity in vitro

**Figure 3.**
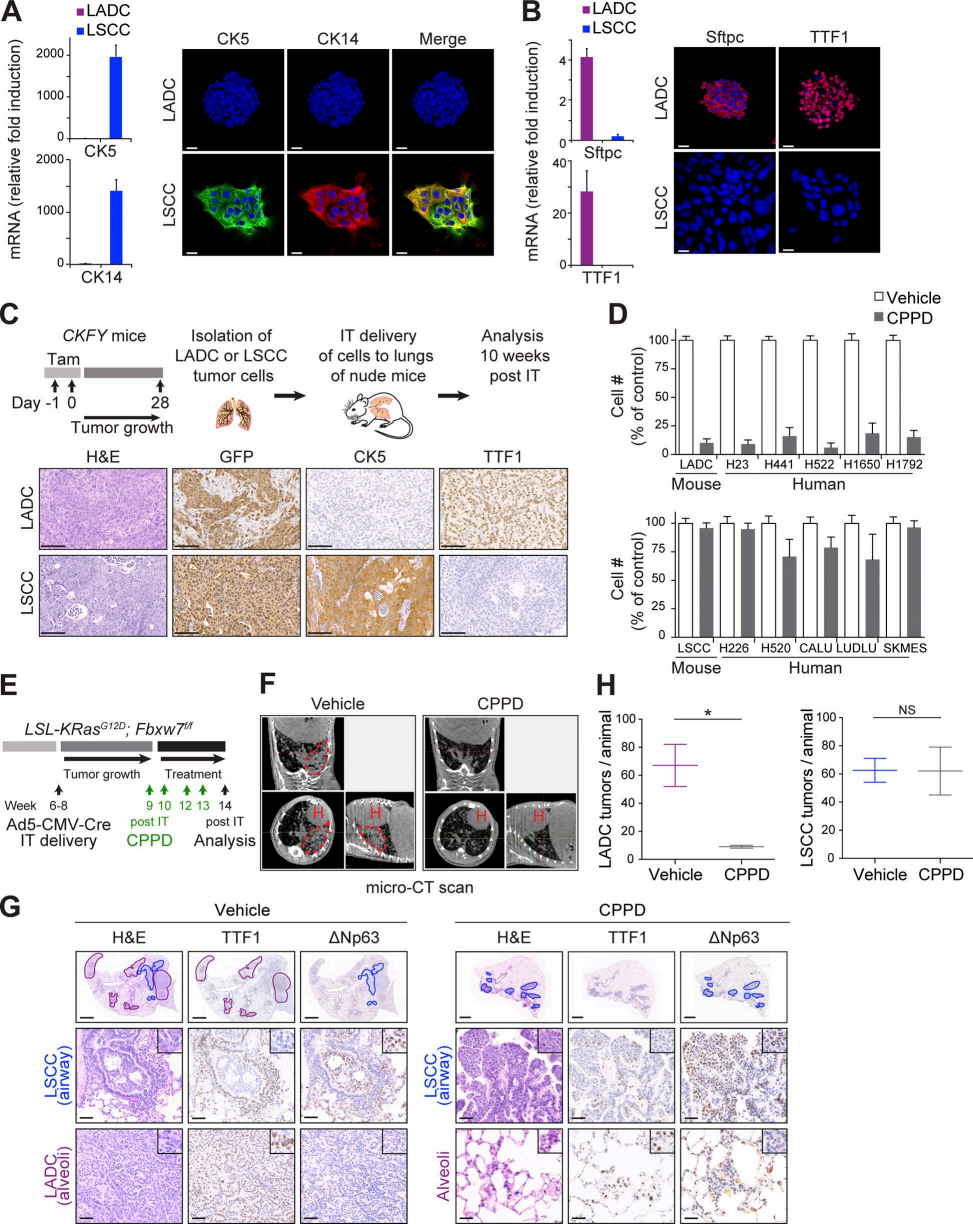
**LSCC tumors are resistant to the chemotherapeutic agent cisplatin. (A)** LADC and LSCC cells were isolated from CKFY mice 4 wk after tamoxifen injection, and CK5 and CK14 expression was analyzed by qPCR and immunostaining. *n* = 2 independent isolations. Bars, 20 µm. **(B)** Sftpc and TTF1 expression in CKFY LADC and LSCC cells (*n* = 2 independent isolations) was analyzed by qPCR and immunostaining. Bars, 20 µm. **(C)** Top: Scheme summarizing the protocol used to isolate LADC and LSCC cells from CKFY mice and to validate their tumoral properties in nude mice. Bottom: Representative IHC staining of lung tumor sections derived from isolated LADC and LSCC tumor cells (*n* = 2 independent isolations) after intratracheal (IT) injection. CKFY-derived, LSCC, and LADC tumor cells were identified by GFP, CK5, and TTF1, respectively. Bars, 100 µm. Tam, tamoxifen. **(D)** LADC, but not LSCC, cells from CKFY mice and human tumor cell lines are highly sensitive to cisplatin (CPPD) treatment (*n* = 4 independent experiments). **(E)** 9–11 wk after Ad5-CMV-Cre infection, KF mice were treated with PBS or cisplatin (7 mg/kg) and monitored for response. **(F)** In vivo imaging by micro-computed tomography (micro-CT) of the lungs of animals treated as in E after 4–5 wk of treatment. Images representative of two animals. H, heart; dotted line, tumor mass. **(G)** Lung histology of animals treated as in E, showing both LADC (TTF1^+^) and LSCC (ΔNp63^+^) tumors in mice receiving vehicle but only LSCC in mice receiving cisplatin. Images representative of two animals. **(H)** Quantification of LADC and LSCC tumors per animal in control and cisplatin-treated mice. *n* = 2 animals per condition. Plots indicate mean and range; Student’s one-tailed *t* test was used to calculate P values (*, P = 0.0305). See also Fig. S4.

Human LADC and LSCC tumors differ not only in their localization and marker expression, but also in their response to therapy. LSCC patients receiving standard platinum-based first-line chemotherapy have shown lower overall survival than LADC patients (Scagliotti et al., 2008; Pirker et al., 2012). The development of two different tumor types in the same genetic model allows a side-by-side comparison of their characteristics without any confounding effects of different driver mutations. With the aid of the YFP lineage tracer and the cell surface markers podoplanin (Schacht et al., 2005) and Sca-1 (Kim et al., 2005), we isolated LSCC and LADC tumor cells, respectively, from CKFY mice by FACS. LADC and LSCC populations appeared morphologically distinct in culture (Fig. S4 A). Whereas LSCC cells proliferated in monolayers and expressed the LSCC-specific markers CK5 and CK14, LADC cells formed aggregates in vitro and expressed Sftpc and TTF1 (Fig. 3, A and B). Complete recombination of *Fbxw7* and *KRas^G12D^* was confirmed by PCR analysis of genomic DNA isolated from both cell types (Fig. S4 B). LSCC, but not LADC, cells in culture expressed a LSCC transcriptional signature similar to that of primary tumor cells from the KF model (Fig. S4 C). Following orthotopic transplantation into immunocompromised mouse recipients, mice that received primary LSCC tumor cells developed tumors with CK5^+^ staining typical of LSCC within 10 wk (Fig. 3 C). In contrast, LADC cells initiated lung tumors expressing the LADC-specific marker TTF1 (Fig. 3 C). Thus, CKFY-derived primary LADC and LSCC tumor cells retain tumor-specific transcriptional signatures and marker protein expression and give rise to distinct tumor types.

### LSCC tumors are resistant to the chemotherapeutic agent cisplatin

To compare the response of LADC and LSCC tumor cells to chemotherapy, we treated them in vitro with 10 µM cisplatin, equivalent to the maximal short-term plasma concentration in patients treated with the drug (Urien and Lokiec, 2004). LADC cells were highly sensitive to cisplatin, but treatment had only a marginal effect on LSCC cell growth (Fig. 3 D). This contrasting behavior of murine KF LADC and LSCC was mirrored in a panel of human lung tumor cell lines, with human LSCC cells showing resistance to cisplatin compared with LADC cells (Fig. 3 D).

We then tested the effect of the maximum tolerated dose of cisplatin (7 mg/kg; Oliver et al., 2010) on LADC and LSCC tumors in vivo, in KF mice infected with Ad5-CMV-Cre virus (Fig. 3 E). In vivo imaging showed reduced lung tumor mass in mice treated with cisplatin compared with vehicle control (Fig. 3 F). On histological inspection, cisplatin-treated lungs showed a significant reduction in LADC lesions, but lesions expressing the LSCC-specific marker ΔNp63 did not show any tumor regression (Fig. 3, G and H), supporting resistance of LSCC cells to chemotherapy.

### LSCC tumors show increased LUBAC expression and linear ubiquitylation

**Figure 4.**
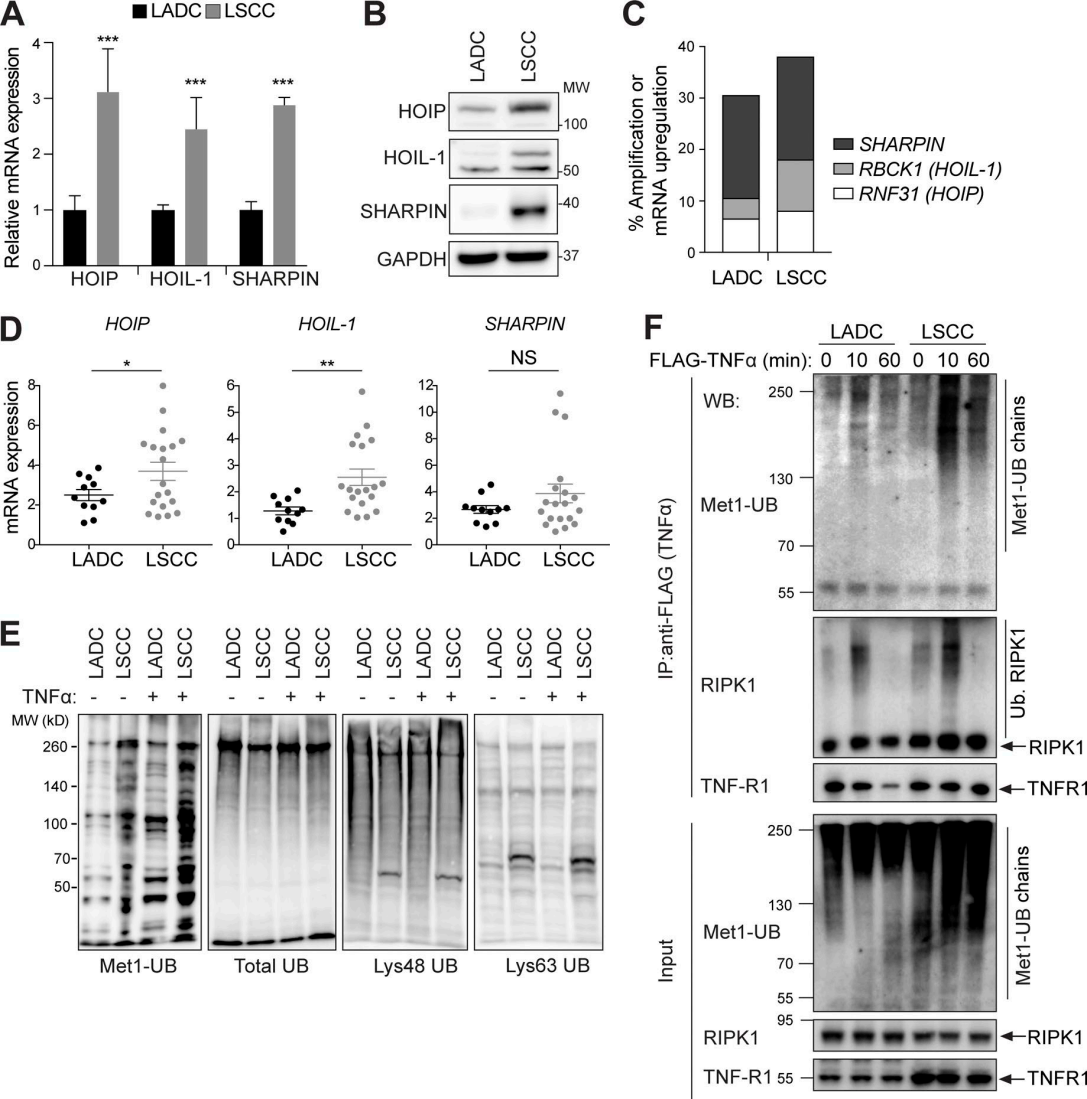
**LUBAC expression and linear ubiquitylation are increased in LSCC compared with LADC cells. (A)** Relative mRNA expression of LUBAC components in LSCC and LADC murine cells from KF mice, measured by real-time PCR. Student’s two-tailed *t* test was used to calculate P values (***, P ≤ 0.0001). Graph shows mean + SD of six experiments. **(B)** Immunoblots showing the abundance of the three LUBAC components HOIP (RNF31), HOIL-1, and Sharpin, in LADC and LSCC cells from the KF mouse model. Blots representative of four independent experiments. MW, molecular weight. **(C)** TCGA data from http://www.cbioportal.org showing frequency of alterations in genes encoding LUBAC components in human LSCC and LADC. *n* = 230 LADC, 179 LSCC samples. **(D)** Relative mRNA expression of LUBAC components in LSCC (*n* = 19) and LADC (*n* = 11) patient samples from the Cordoba Biobank, measured by real-time PCR. The P value was calculated using the Student’s *t* test (*, P = 0.036; **, P = 0.0029). Plots indicate mean ± SEM. **(E)** Immunoblots of different polyubiquitin chains in whole-cell lysates from untreated (no exogenous stimulation) or TNFα-stimulated LADC and LSCC cells from KF mice. **(F)** TNFR1 complex I immunoprecipitated (IP) from KF LADC and LSCC cells using Flag-tagged TNFα under nondenaturing conditions and immunoblotted with Met1-Ub–specific antibodies. Blots representative of two independent experiments. WB, Western blot. See also Fig. S4.

To understand the molecular basis of the difference in cisplatin sensitivity between LADC and LSCC tumors, we examined mRNA and protein expression in KF LADC and LSCC tumor cells. The levels of known FBW7 substrates, including MCL1, c-Jun, NICD1 (inferred from transcription levels of its target genes), and c-Myc, which all have reported roles in cell survival (Davis et al., 2014), were not significantly different in LADC versus LSCC cells (Fig. S4, D and E). In contrast, our transcriptome data revealed that HOIP (*Rnf31*), HOIL-1 (*Rbck1*), and Sharpin, components of LUBAC, were more highly expressed in LSCC compared with LADC tumor cells, which was confirmed by quantitative PCR (qPCR) and immunoblotting (Fig. 4, A and B). In agreement with these findings, analysis of TCGA data showed that amplification or mRNA up-regulation of the genes encoding LUBAC components was more common in human LSCC compared with LADC (Fig. 4 C and Fig. S4 F). We examined the expression of LUBAC components in 30 primary human lung tumor samples and confirmed increased expression of *HOIL-1* and *HOIP* mRNA in LSCC compared with LADC (Fig. 4 D).

Consistent with increased LUBAC expression, LSCC cell lysates showed increased Met1-linked ubiquitylation, which further increased following TNFα stimulation (Fig. 4 E). The increase in linear ubiquitylation was not due to an overall change in total ubiquitin species or seen with other ubiquitin chain topologies (Fig. 4 E). Moreover, capture of intact TNFR1 complex-I using FLAG-tagged TNFα and immunoblotting with Met1 linkage-specific antibodies showed that linear ubiquitylation of complex-I proteins was also increased in LSCC compared with LADC cells (Fig. 4 F). Together, our data indicate that LSCC have higher levels of LUBAC and linear ubiquitylation.

### LSCC tumors show increased NF-κB signaling

**Figure 5.**
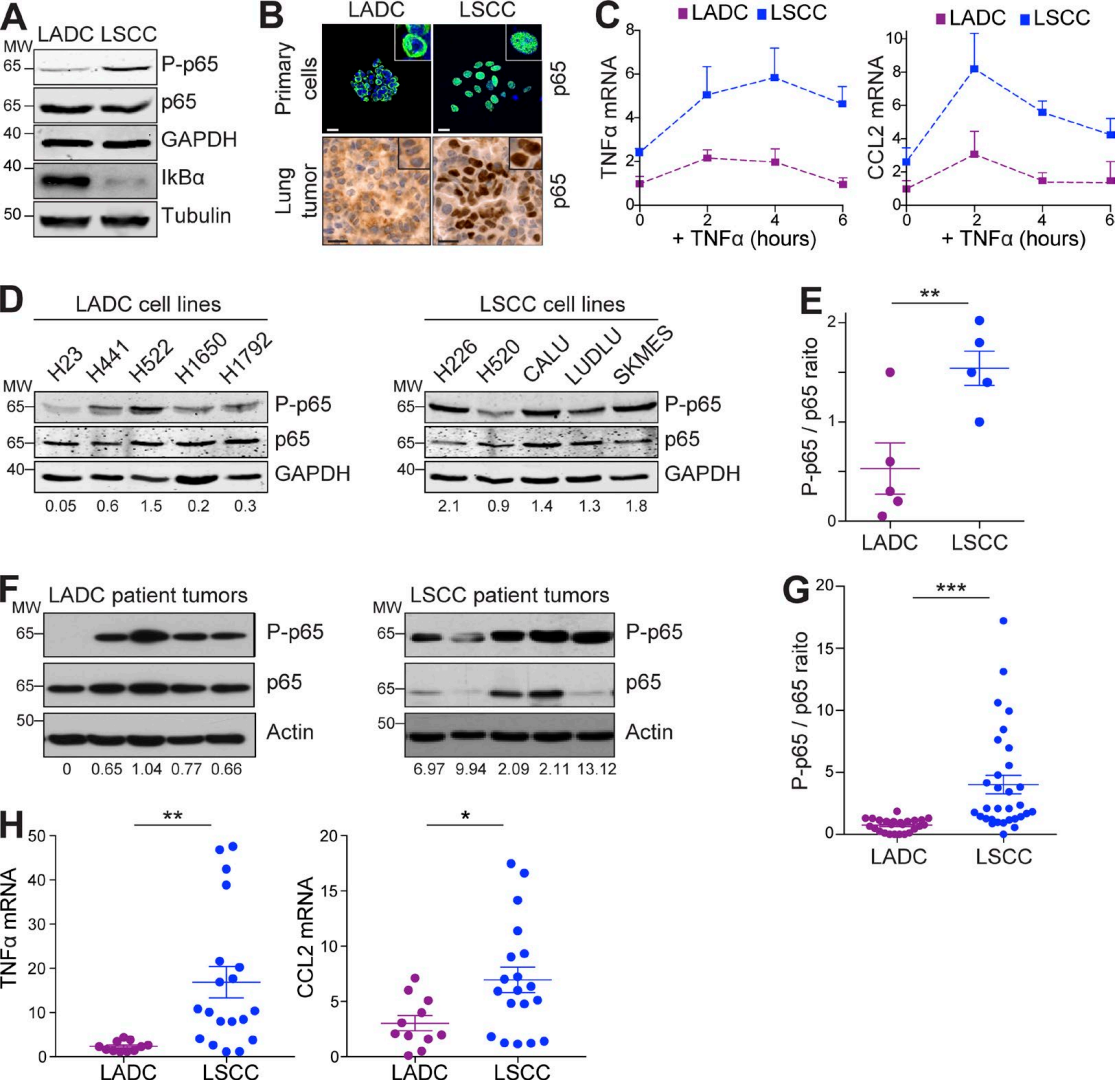
**Mouse and human LSCC tumors show activated NF-κB signaling. (A)** Western blots showing LSCC cells from KF mice have a higher baseline level of phospho-p65 and a lower level of IκBα. Blots representative of two independent experiments. MW, molecular weight. **(B)** Immunofluorescent staining on LADC and LSCC primary tumor cells (top, representative of *n* = 3 independent isolations) and IHC staining of LADC and LSCC tumors (bottom, representative of *n* = 3 animals) from KF mice showing increased nuclear localization of p65 in LSCC. **(C)** LSCC cells have a higher baseline level of NF-κB–dependent target gene (*CCL2* and *TNFα*) expression and respond more following TNFα stimulation when compared with LADC cells. Relative mRNA levels were measured by real-time PCR following TNFα stimulation (10 ng/ml) of LADC and LSCC cells for the indicated times. Graph represents mean (+ SD) of three independent experiments performed in duplicate. **(D)** Western blots showing that human LSCC tumor cell lines exhibit higher levels of p65 activation compared with LADC cell lines. Ratio of phospho-p65 to total p65 normalized to GAPDH is given below. Samples were run in parallel, and identical exposures were used. **(E)** Quantification of results in D. Student’s one-tailed *t* test was used to calculate P values (**, P = 0.0095). Plots indicate mean ± SEM. **(F)** Human LSCC tumors exhibit higher levels of p65 activation compared with LADC samples. Ratio of phospho-p65 to total p65 normalized to actin is given below. Samples were run in parallel, and identical exposures were used. **(G)** Quantification of results in F and from a total of 25 LADC and 31 LSCC patient tumors. Student’s two-tailed *t* test was used to calculate P values (***, P = 0.0002). Plots indicate mean ± SEM. **(H)** Relative mRNA expression of CCL2 and TNFα in LSCC (*n* = 19) and LADC (*n* = 11) patient samples from the Cordoba Biobank, measured by real-time PCR. Student’s two-tailed *t* test was used to calculate P values (*, P = 0.01; **, P = 0.0049). Plots indicate mean ± SEM. See also Fig. S4.

To determine whether the increased linear ubiquitylation seen in LSCC cells was associated with increased NF-κB signaling, we examined levels of the NF-κB inhibitor IκBα and Ser536 phosphorylation of the NF-κB subunit p65 (P-p65) in LADC and LSCC cells. LSCC cells had lower levels of IκBα and higher levels of P-p65 compared with LADC cells, suggesting active NF-κB (Fig. 5 A). In line with this, p65 showed strong nuclear localization in LSCC but not in LADC tumor cells (Fig. 5 B). Correspondingly, basal expression of the NF-κB–dependent target genes TNFα and CCL2 was higher in LSCC than LADC cells, and was further increased by TNFα stimulation (Fig. 5 C). LSCC cells, both in culture and from primary tumors, also showed up-regulation of multiple NF-κB target genes compared with LADC cells (Fig. S4 G).

The differences in NF-κB activation between LADC and LSCC were conserved in human LADC and LSCC cell lines (Fig. 5, D and E), and this finding was further confirmed in a panel of primary human LSCC tumor samples. Protein extracts from 25 human LADC and 31 human LSCC freshly isolated tumor resections were generated, and p65 phosphorylation was analyzed (representative examples in Fig. 5 F). Quantification of P-p65/total-p65 ratio revealed substantially increased P-p65 levels in human LSCC, and expression of CCL2 and TNFα was correspondingly increased (Fig. 5, G and H). These data suggest that a high level of NF-κB activation is a feature of LSCC, both in the KF mouse model and human LSCC patients.

### Increased NF-κB signaling in LSCC tumors mediates cisplatin resistance

**Figure 6.**
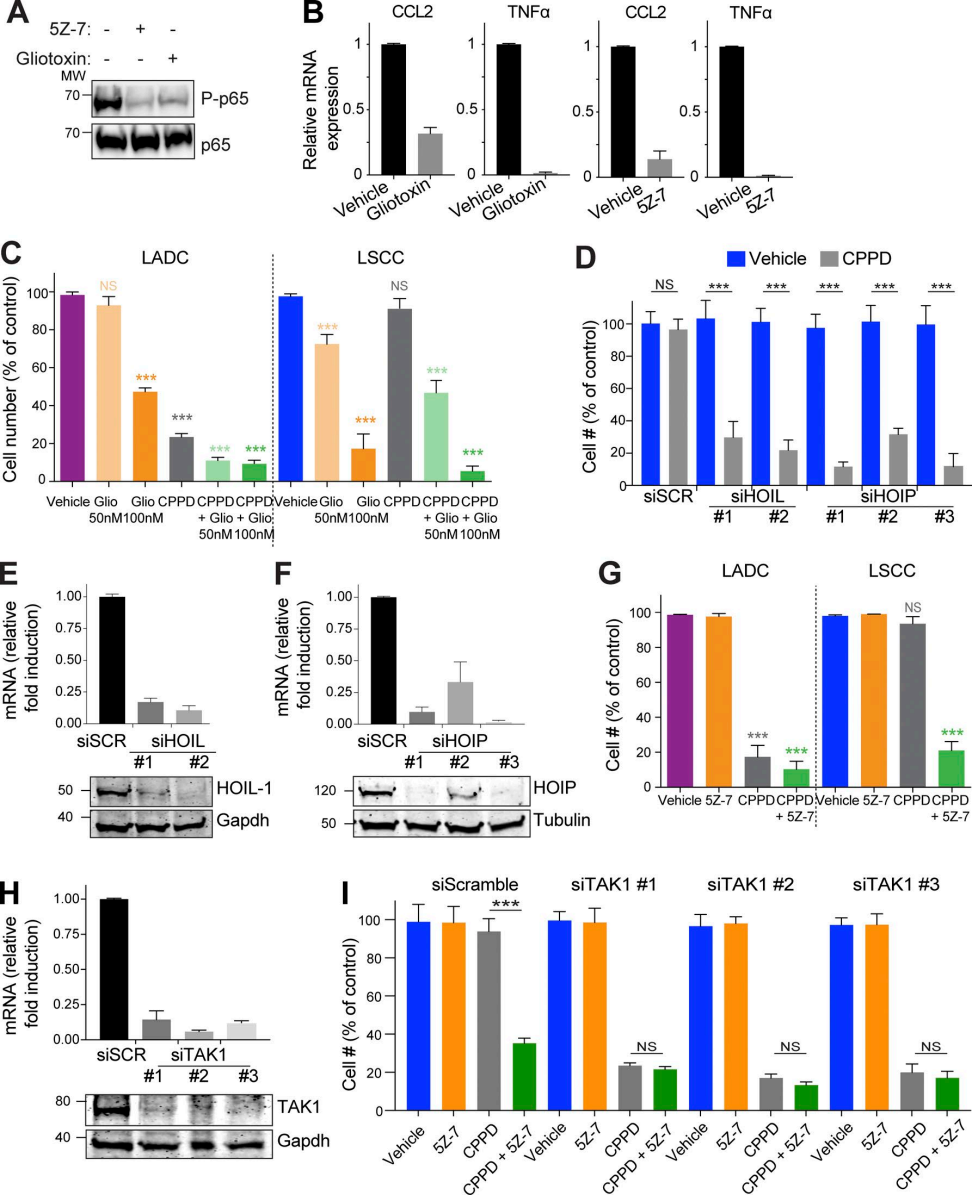
**Inhibition of LUBAC or TAK1 sensitizes LSCC cells to cisplatin. (A)** Both 5Z-7 and gliotoxin inhibit NF-κB signaling, as measured by phospho-p65 (Ser536). Blots represent two independent experiments. MW, molecular weight. **(B)** Expression of NF-κB–dependent target genes (*CCL2* and *TNFα*) in murine LSCC cells is decreased in the presence of the TAK1 inhibitor 5Z-7 or the LUBAC inhibitor gliotoxin. Relative mRNA levels measured by real-time PCR. Data are mean + SD of three independent experiments performed in triplicate. **(C)** LSCC cells are highly sensitive to gliotoxin treatment, and gliotoxin sensitizes cells to cisplatin. Data are mean + SD of two independent experiments performed in duplicate. ***, P < 0.0001 versus vehicle, two-way ANOVA. **(D)** siRNA-mediated knockdown of the LUBAC components HOIP and HOIL-1 decreases LSCC cell resistance to cisplatin. Graph shows mean + SD of three independent experiments performed in triplicate. ***, P < 0.0001 versus vehicle, one-way ANOVA. **(E)** Validation of HOIL-1 knockdown in LSCC cells using two independent siRNAs. Graphs represent mean + SEM of three independent experiments. **(F)** Validation of HOIP knockdown in LSCC cells using three independent siRNAs. Graphs represent mean + SEM of three independent experiments. **(G)** In vitro LSCC and LADC cells are highly sensitive to cisplatin (CPPD) and 5Z-7-oxozeaenol combination treatment. Data are mean + SD of two independent experiments performed in duplicate. ***, P < 0.0001 versus vehicle, two-way ANOVA. **(H)** Validation of TAK1 knockdown in LSCC cells using three independent siRNAs. Graphs represent mean + SEM of three independent experiments. **(I)** siTAK1 LSCC cells are sensitive to cisplatin (CPPD), and addition of 5Z-7 does not affect CPPD sensitivity in the presence of TAK1 knockdown. P values calculated using one-way ANOVA test. Graph shows mean + SD of two independent experiments performed in quadruplicate. ***, P < 0.0001 versus vehicle, one-way ANOVA. See also Fig. S5.

To assess the importance of LUBAC and NF-κB activity in the chemotherapy resistance of LSCC, we treated primary KF LSCC tumor cells with gliotoxin, which inhibits LUBAC by direct binding to HOIP (Sakamoto et al., 2015), or with a TAK1 inhibitor, 5Z-7-oxozeaenol (5Z-7), which is required for activation of IKK and IKK-mediated degradation of IκB (Wang et al., 2001). Both gliotoxin and 5Z-7 decreased NF-κB activity, as measured by P-p65 and expression of CCL2 and TNFα (Fig. 6, A and B). LSCC cells were more sensitive to gliotoxin than LADC cells, and gliotoxin sensitized LSCC cells to cisplatin, suggesting that the chemoresistance of LSCC tumor cells is mediated, at least in part, by LUBAC (Fig. 6 C). In support of this idea, knockdown of the LUBAC components HOIL-1 or HOIP also sensitized LSCC cells to cisplatin (Fig. 6, D–F). Treatment with 1 µM 5Z-7 strongly sensitized murine LSCC cells to cisplatin (Fig. 6 G; combination index [CI] 0.39, indicating synergistic activity). The combination of 5Z-7 and cisplatin was more effective than cisplatin alone in four of five human LSCC cell lines (Fig. S5 A). Cisplatin did not have an additional effect on inhibition of P-p65 by 5Z-7 (Fig. S5 B). Since both human and mouse LADC cells were already sensitive to cisplatin alone, the additional effect of the combination treatment was less striking in LADC (Fig. 6 G and Fig. S5 A). To confirm that the effect of 5Z-7 was via inhibition of TAK1, we knocked down TAK1 in LSCC cells. Like 5Z-7, *siTAK1* sensitized LSCC cells to cisplatin, and addition of 5Z-7 did not significantly increase sensitivity (Fig. 6, H and I). Inhibition of the TAK1 target and NF-κB upstream regulator IKK also sensitized LSCC cells to cisplatin (Fig. S5 C).

### LUBAC or TAK1 inhibition sensitizes LSCC tumors to cisplatin chemotherapy in preclinical models

**Figure 7.**
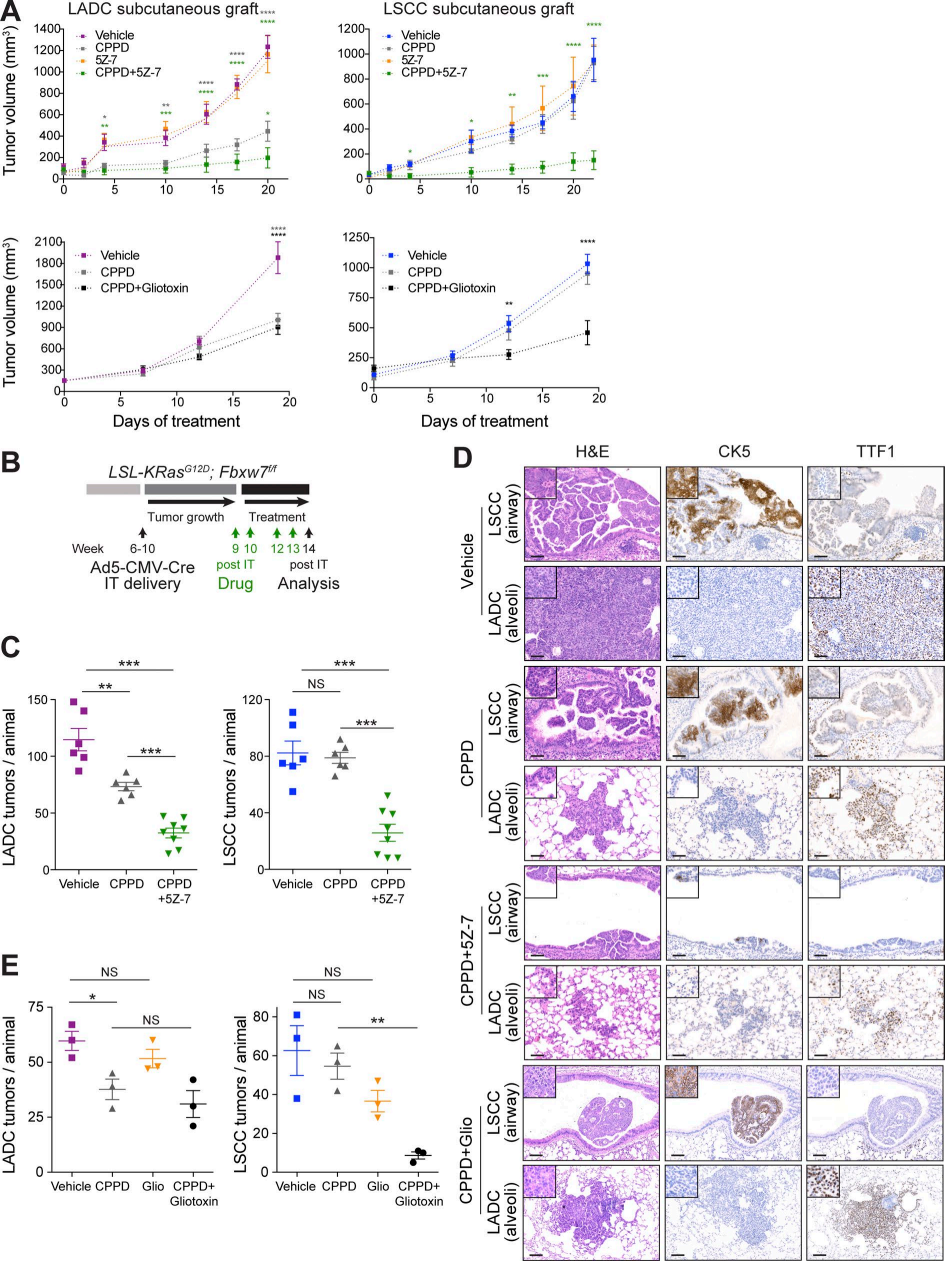
**Combination treatment with cisplatin and TAK1 inhibitor or gliotoxin sensitizes LSCC tumors. (A)** In vivo tumor graft growth curves of LADC and LSCC cells subcutaneously injected in both flanks of athymic NU/NU mice. Mice with palpable tumors were treated with i.p. injections as follows. Top: Cisplatin (CPPD; *n* = 3), 5Z-7-oxozeaenol (*n* = 3), 5Z-7-oxozeaenol+cisplatin (*n* = 3), or vehicle (*n* = 3). Bottom: Cisplatin (*n* = 3), gliotoxin+cisplatin (*n* = 4), or vehicle (*n* = 4). Data are mean ± SEM of the tumor volumes. P values calculated from two-way ANOVA. *, P < 0.05; **, P < 0.01; ***, P < 0.001; ****, P < 0.0001 versus vehicle. **(B)** Scheme depicting experimental design for in vivo test of cisplatin (3.5 mg/kg) alone or in combination with 5Z-7-oxozeaenol (7 mg/kg) or gliotoxin (2.5 mg/kg). **(C)** Quantification of LADC and LSCC tumors per animal in control, cisplatin-treated, and 5Z-7 combination-treated KF mice. P values calculated using two-way ANOVA (*n* = 6 vehicle and cisplatin, *n* = 8 combination). **, P = 0.009; ***, P < 0.001. Plots show mean ± SEM. **(D)** Histological analysis of LADC and LSCC tumors in control, cisplatin-treated, and combination-treated animals. Representative of animals in C (vehicle, cisplatin, and 5Z-7 combination) and E (gliotoxin combination). Bars, 50 µm. **(E)** Quantification of LADC and LSCC tumors per animal in control, cisplatin-treated, and gliotoxin combination–treated KF mice. P values calculated using two-way ANOVA (*n* = 3 each treatment; *, P = 0.031; **, P = 0.0013). Plots show mean ± SEM. See also Fig. S5.

Next, we investigated the effect of combining cisplatin with inhibition of the TAK1-LUBAC/IKK-NF-κB axis in both subcutaneous lung tumor grafts and autochthonous lung cancer models. Pilot experiments suggested that the combination of maximal tolerated doses of cisplatin (7 mg/kg) and 5Z-7 (15 mg/kg) or gliotoxin (5 mg/kg) was not well tolerated (mice experienced >15% weight loss), so we used half doses of both drugs in all combination-treatment experiments. The combination of 3.5 mg/kg cisplatin and 7 mg/kg 5Z-7 strongly suppressed the growth of subcutaneous lung tumor grafts (Fig. 7 A). Cisplatin combined with 2.5 mg/kg gliotoxin had a similar effect (Fig. 7 A).

To validate the therapeutic efficacy of the combination therapy in a preclinical lung tumor model, we used KF mice infected with Ad5-CMV-Cre virus (Fig. 7 B). 9 wk after infection, when mice had developed lung tumors, we started treatment. As observed with the 7 mg/kg cisplatin dose (Fig. 3 H), treatment with 3.5 mg/kg cisplatin alone reduced exclusively LADC tumors, with LSCC tumors being nonresponsive (Fig. 7 C). However, combination treatment with 5Z-7 sensitized LSCC tumors to cisplatin and induced a significant reduction in LSCC tumor number (Fig. 7, C and D). IHC analysis of Ki67 and active caspase-3 in tumors treated with cisplatin plus 5Z-7 showed decreased cell proliferation and increased cell death compared with vehicle-treated tumors (Fig. S5, D and E). TAK1 inhibition also increased cisplatin efficacy toward LADC tumors (Fig. 7, C and D), albeit incrementally.

Using the same experimental setup, we also tested the combination of gliotoxin plus cisplatin in tumor-bearing KF mice. Although neither gliotoxin nor cisplatin had a significant effect on LSCC tumor burden alone, the combination treatment reduced LSCC tumor number by >80% (Fig. 7 E). The remaining tumors also showed strongly reduced Ki67 positivity (Fig. S5 D). Interestingly, unlike 5Z-7, addition of gliotoxin did not significantly increase cisplatin sensitivity in LADC tumors, making the combination treatment effect with gliotoxin specific to LSCC (Fig. 7 E).

This experimental setting also allowed us to monitor potential side effects that could result from the combination treatments. Animals tolerated the drug combinations well, with no significant loss of body weight (Fig. S5 F). Importantly, given the risk of renal toxicity with cisplatin chemotherapy (Sahni et al., 2009), we found no evidence of morphological changes or fibrosis in the kidneys of combination therapy–treated versus vehicle-treated animals (Fig. S5 G). Immune infiltration of CD4^+^ T cells and macrophages, which plays a role in the development of cisplatin-induced kidney injury, was not significantly different between the treatment groups, and there was no evidence of cell death in the kidney (Fig. S5 H). Thus, combination therapy with 3.5 mg/kg cisplatin and 7 mg/kg 5Z-7 or 2.5 mg/kg gliotoxin is well tolerated by adult mice.

**Figure 8.**
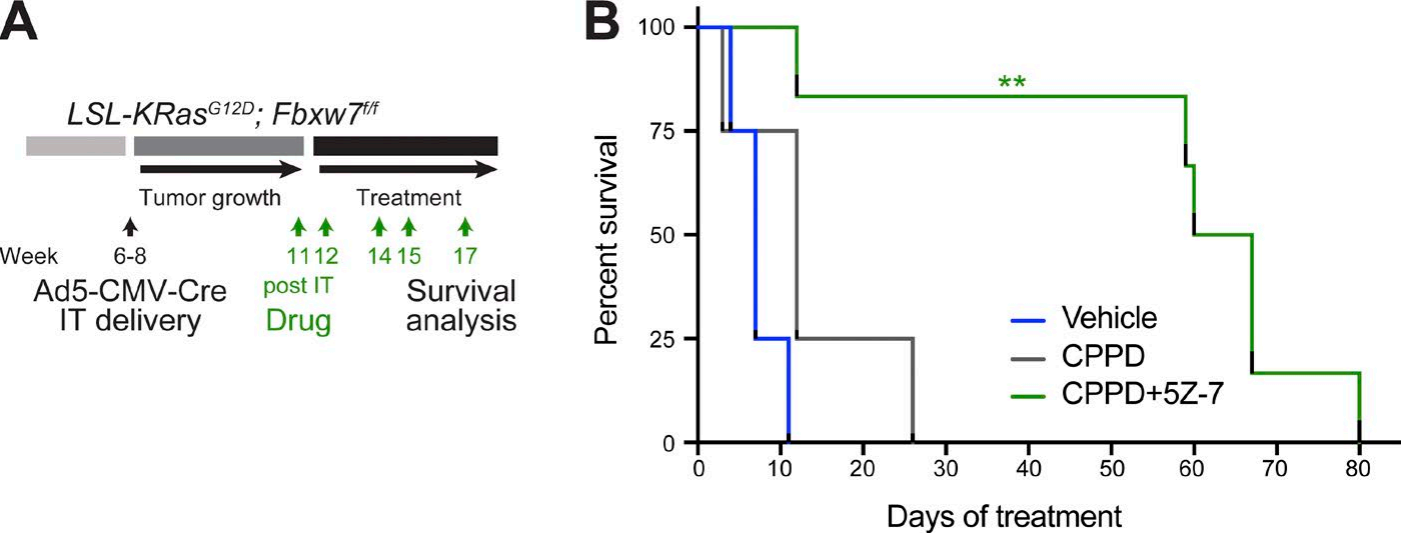
**Combination treatment with cisplatin and TAK1 inhibitor increases survival even with advanced tumors. (A)** Scheme depicting experimental design for end-stage survival analysis of control, cisplatin-treated, and combination-treated KF mice. IT, intratracheal. **(B)** Kaplan-Meier curve showing effect of cisplatin (CPPD; 3.5 mg/kg) or cisplatin plus 5Z-7-oxozeaenol (7 mg/kg) treatment on survival of tumor-bearing KF mice (*n* = 4 vehicle and cisplatin, *n* = 6 combination). P value calculated using log-rank (Mantel–Cox) test (**, P = 0.0011). See also Fig. S5.

To interrogate the therapeutic effect of combination treatment in advanced lung cancer, we treated KF mice with a very high lung tumor load (Fig. 8 A). Vehicle- and cisplatin-treated KF mice all had to be sacrificed within 1–2 wk of treatment onset (median survival of 7 and 12 d, respectively). The combination of cisplatin and 5Z-7 treatment resulted in a significant increase in survival, with five of six animals surviving for >7 wk (Fig. 8 B). Because gliotoxin/cisplatin combination therapy was more effective on LSCC than LADC tumors (Fig. 7 E), we did not test this combination in late-stage KF mice, since both tumor types would need to be controlled to achieve survival benefit. Nevertheless, this experiment suggests that 5Z-7/cisplatin combination therapy could be of therapeutic benefit for lung cancer patients with advanced disease.

Overall, these data identify LUBAC levels, and NF-κB activity, as a determinant of chemotherapy resistance in squamous lung tumors and suggest LUBAC or TAK1 inhibitors in combination with platinum-based drugs as a possible therapy for LSCC.

## Discussion

Here, we show that inactivation of *Fbxw7* concomitant with *KRas^G12D^* expression in the adult mouse lung (KF model) leads to both LSCC and LADC formation. Using lung cell type–restricted Cre viruses, we showed that KF LADC tumors originate mainly from alveolar Sftpc^+^ cells, but that targeted inactivation of *Fbxw7* and activation of *KRas^G12D^* in club CC10^+^ cells generated predominantly LSCC lesions. In contrast, basal cells did not give rise to LSCC tumors in this mouse model. As observed in human patients, KF LSCC tumors were located in, and adjacent to, the airways and expressed biomarkers characteristic of human LSCC, such as CK5 and ΔNp63. During LSCC progression, lesions lost CC10 and gained CK5 expression. Given FBW7’s role in regulating cell differentiation, it is possible that *Fbxw7* inactivation (in our KF model) or loss of FBW7 protein (in human LSCC) causes some CC10^+^ cells to lose their identity and acquire a squamous phenotype.

Activation of *KRas^G12D^*, with or without concomitant p53 inactivation (KP model), induces LADC in both CC10^+^ and Sftpc^+^ populations, with the tumors being located predominantly in the alveolar space (Sutherland et al., 2014). We made similar observations regarding LADC development in the KF model. Sftpc^+^ cells and CC10^+^ cells generated LADC lesions; however, in addition CC10^+^ cells efficiently originated LSCC. Sutherland et al. (2014) reported that although *KRas^G12D^* was activated in many CC10^+^ cells that line the bronchi and bronchioles, they rarely observed tumor formation in this area. Thus, CC10^+^ cells located near the airways appear to be susceptible to the KF, but not to the KP oncogene combination.

The cell of origin of LSCC has been recently studied in several mouse models, with different results. Depending on the nature of the driver mutations, either basal, club, or alveolar type II cells had the capability to give rise to LSCC (Xu et al., 2014; Ferone et al., 2016; Jeong et al., 2017). These findings suggest that different cell types can be lung LSCC-initiating cells when supplied with the right set of oncogenic triggers.

Unlike LADC, there are few approved targeted therapies against LSCC. Consequently, despite its limited effectiveness on disease progression and prognosis, patients with LSCC receive the same conventional platinum-based chemotherapy today as they would have received two decades ago (Scagliotti et al., 2008; Liao et al., 2012; Gandara et al., 2015; Fennell et al., 2016; Isaka et al., 2017).

We identified increased LUBAC expression and linear ubiquitylation in LSCC tumors, correlating with activation of NF-κB signaling in human and mouse LSCC. Notably, all five human LSCC cell lines tested showed increased p65 phosphorylation and were resistant to cisplatin (Fig. 5 D and Fig. 3 D), despite the fact that in at least three of these cell lines, *FBXW7* is not mutated (Forbes et al., 2017). Moreover, the same KF driver oncogene combination which resulted in cisplatin-resistant LSCC failed to induce cisplatin resistance in LADC cells. Therefore, high NF-κB activity, and consequently cisplatin resistance, is unlikely to be a direct molecular consequence of the absence of FBW7, but rather a general property of LSCC tumor cells.

Both murine and human LSCC tumor cells were resistant to cisplatin, and inhibition of NF-κB signaling using either gliotoxin or 5Z-7-oxozeaenol resulted in sensitization of LSCC tumor cells to cisplatin chemotherapy, suggesting a crucial role of increased NF-κB signaling in the chemotherapy response of LSCC. Importantly, our results show that using a half dose of cisplatin in combination with gliotoxin or 5Z-7-oxozeaenol is sufficient to decrease proliferation and impair LSCC tumor growth. As a consequence, tumor-bearing mice survived significantly longer than control and cisplatin-treated animals. It is important to note that mice did not show significant weight loss or renal toxicity. Thus, our combination strategy provides significant therapeutic benefit.

In summary, our study demonstrates that the TAK1-LUBAC/IKK-NF-κB axis is a key mediator of LSCC progression and hence represents a suitable therapeutic target. Therefore, LUBAC or NF-κB inhibition combined with conventional chemotherapy should be considered as a potential therapy for human LSCC.

## Materials and methods

### Analysis of TCGA data

Data from TCGA Research Network (TCGA Lung Adenocarcinoma and Lung Squamous Cell Carcinoma Provisional complete sample sets), including mutations, putative copy-number alterations, and mRNA z-scores (RNA Seq V2 RSEM; threshold 2.0), were analyzed using cBioportal software (Cerami et al., 2012) and visualized using the standard Oncoprint output.

### Human lung tumor analysis

Human biological samples used in the current study were collected, stored, and managed by the Cordoba node belonging to the Biobank of the Andalusian Health Service (Servicio Andaluz de Salud-SAS) and approved by the Ethics and Clinical Research Committee of the University Hospital Reina Sofia. All subjects gave informed consent. Pathologists assessed all samples before use. Human lung samples were stained for FBW7 expression, and protein lysates were analyzed for phospho-p65, p65, and actin by Western blotting. mRNA extracted from the samples was analyzed by qPCR. Primers and antibodies are listed in Tables S1 and S3.

### Mouse strains

The *KRas^G12D^* (Jackson et al., 2001), *R26-LSL-YFP* (Srinivas et al., 2001), *CK5-Cre^ERT^* (Indra et al., 1999), *CK14-Cre^ERT^* (Vasioukhin et al., 1999), *CK19-Cre^ERT^* (Means et al., 2008), and *Fbxw7^f/f^* (Jandke et al., 2011) mouse lines have been previously described. All animal experiments were approved by the Francis Crick Institute Animal Ethics Committee and conformed to UK Home Office regulations under the Animals (Scientific Procedures) Act 1986 including Amendment Regulations 2012.

### Genetic labeling experiments

For all experiments, adult (6–9 wk) age- and strain-matched animals were used. Mice were injected i.p. with 100 µg/kg body weight of tamoxifen dissolved in peanut oil for a consecutive 2–5 d. Analyses were performed at different time points after injection. Where indicated, EdU (50 mg/kg) was given i.p. 10 h before the end of the experiment.

### Generation of recombinant cell type–specific Cre viruses

Human FoxJ1 promoter-Cre and bovine cytokeratin 5 promoter-Cre constructs have been previously described (Ramirez et al., 2004; Zhang et al., 2007) and were subcloned either in LV-PL1 as SpeI–BsrGI or in pAd5mcspA as NotI–NotI, respectively. Ad5-CC10-Cre, Ad5-CMV-Cre, and Ad5-SPC-Cre have been previously described (Sutherland et al., 2011). High-titer viruses were amplified and purified for use in vivo by the University of Iowa Gene Transfer Vector Core, supported in part by the National Institutes of Health and the Roy J. Carver Foundation, for viral vector preparation.

### Intratracheal Adeno-Cre or Lenti-Cre virus administration

6–8-wk-old mice were intratracheally intubated with 50 µl of purified Cre viruses: Ad5-CMV-Cre, Ad5-SPC-Cre, Ad5-CC10-Cre, and Ad5-CK5-Cre were used at 2.5 × 10^7^ PFU; Lenti-FoxJ1-Cre was used at 1.4 × 10^8^ PFU. To target basal CK5^+^ cells, mice were administered naphthalene (250 mg/kg) 3 d before Ad5-CK5-Cre infection. Ad5-CK5-Cre–calcium phosphate coprecipitates were prepared according to published methods (DuPage et al., 2009).

### Histology, IHC, and immunofluorescence

For histological analysis, lungs were fixed overnight in 10% neutral buffered formalin. Fixed tissues were subsequently dehydrated and embedded in paraffin, and sections (4 µm) were prepared for H&E staining, IHC, or immunofluorescence. Antibodies are given in Table S3. To quantify EdU^+^ cells (stained using Click-iT EdU Alexa Fluor 647 Imaging kit; C10340; Thermo Fisher Scientific), 25 airways were scored from at least three mice. For quantification of Ki67, C3A, CD4, and F4/80, 15–30 representative sections were analyzed with MetaMorph 6.1 or ImageJ-IHC tool box software. Data are represented as mean ± SEM.

### Isolation of tumor cells by FACS

Single lung cell suspensions were obtained by 20-min digestion in a mix of collagenase (0.5–3 mg/ml; Worthington) and Dispase (1 mg/ml; Invitrogen), followed by filtration through cell strainers (100, 70, and 40 µm; Ruiz et al., 2014). Cells from CKFY mice were first sorted for GFP expression and then sorted for Sca1 (LADC cells) or podoplanin (LSCC cells), using antibodies listed in Table S3. The isolated cells were cultured in N2B27 medium containing EGF (10 ng/ml; Pepro Tech) and FGF2 (20 ng/ml; Pepro Tech).

### RNASeq/gene expression profile analysis

LSCC and LADC cells were isolated as explained above. RNA was isolated using the MagMAX-96 total RNA Isolation Kit, following the manufacturer’s instructions (Thermo Fisher Scientific). Read mapping and abundance estimation were performed using RSEM v1.2.31 (Li and Dewey, 2011) running STAR v2.5 (Dobin and Gingeras, 2015) against the *Mus musculus* Ensembl 86 transcriptome (GRCm38), with the following nondefault command line arguments: “–star–forward-prob 0.” Further analysis was conducted using R v3.4.0 running Bioconductor v3.5. Gene-level read count estimates were rounded to integer values and used by DESeq2 (Love et al., 2014) to assess differential expression between LSCC and LADC replicate groups using the “DESeq2” function with default settings.

Normalized counts were used to construct an expression heatmap for LSCC signature genes common to two previous studies (Xu et al., 2014; Ferone et al., 2016). For the purposes of visualization, gene counts across samples were converted to z-scores and ordered by magnitude of change. 

RNASeq data were deposited in the Gene Expression Omnibus (GEO) repository under accession no. GSE123716.

### DNA isolation and allele recombination PCR

To verify Cre-mediated recombination of *KRas^G12D^* and *Fbxw7* alleles, genomic DNA from cells was isolated by digestion in DirectPCR Lysis Reagent (Viagen Biotech). PCR primers used to detect the efficiency of recombination of *Fbxw7* and *KRas^G12D^* alleles are given in Table S2.

### Gene expression analysis

RNA was isolated from sorted cells using an RNeasy Micro Kit (Qiagen), and cDNA amplification was performed using the Transcriptor First Strand cDNA Synthesis Kit (Roche). Diluted cDNAs were used for quantitative real-time PCR SYBR-Green detection of target genes, using primer sequences given in Table S1.

### Immunoblot analysis

Cells were lysed in ice-cold lysis buffer (20 mM Tris HCl, pH 7.5, 5 mM MgCl_2_, 50 mM NaF, 10 mM EDTA, 0.5 M NaCl, and 1% Triton X-100) that was completed with protease, phosphatase, and kinase inhibitors. Antibodies are given in Table S3.

### Cell proliferation assay

Murine and human tumor cell lines were seeded at 2 × 10^3^ and 4 × 10^3^, respectively, in 96-well plates and, 16 h later, were treated with vehicle, cisplatin (10 µM), 5Z-7-oxozeaenol (1 µM), or the combination. Cell confluence was measured over several days, using an incubator microscope system for live cell imaging (IncuCyte), and used as the readout for cell proliferation. Alternatively, after 5 d, the cells were trypsinized and counted using a hemocytometer.

### In vivo pharmacology with subcutaneous graft tumors

Murine LADC and LSCC tumor cells were resuspended as single-cell suspensions at 10^7^ cells/ml in PBS:Matrigel. 100 µl (10^6^ cells total) of this suspension was injected into opposite left (LADC) and right (LSCC) flanks of athymic NU/NU nude mice. Two weeks later when tumors were palpable, treatment with gliotoxin (2 mg/kg), cisplatin (3.5 mg/kg), and 5Z-7-oxozeaenol (7 mg/kg) was initiated. Mice were treated every 5 d for a total of four doses. Tumor grafts were measured with digital calipers, and tumor volumes were determined with the following formula: (length × width^2^) × (π/6). Tumor volumes are plotted as means ± SEM.

### Cisplatin and 5Z-7-oxozeaenol treatment

9–11 weeks after Ad5-CMV-Cre infection, *LSL-KRas^G12D^; Fbxw7^f/f^* mice were treated with vehicle, cisplatin alone (3.5 or 7 mg/kg), or the combination of cisplatin (3.5 mg/kg) and 5Z-7-oxozeaenol (7 mg/kg) or gliotoxin (2.5 mg/kg) once a wk for 2 wk, followed by a 1-wk break, and this regimen was repeated for a total of four doses before histological analysis or until humane survival endpoint was reached.

### HOIL, HOIP, and TAK1 genetic silencing

LSCC cells were transfected with specific siRNAs against the HOIL*/Rbck1*, HOIP*/Rnf31*, and TAK1*/Map3k7* genes (sequences in Table S2), using Lipofectamine RNAiMAX and 25 nM of each siRNA according to the manufacturer’s instructions (Dharmacon). 96 h later, cells were treated with cisplatin (10 µM) or 5Z-7 (1 µM), and after 3–4 d, cell viability was measured as the intracellular ATP content using the CellTiter-Glo Luminescent Cell Viability Assay (Promega), following the manufacturer’s instructions.

### CI assays

For drug combination experiments, LSCC cells were seeded in 96-well plates at a density of 2 × 10^3^ cells per well, and TAK1 inhibitor+cisplatin combination was added for 3–4 d, using DMSO as a control. Cell viability was measured as explained above. The CI was calculated using CalcuSyn software (Biosoft) following the method of Chou and Talalay (1983).

### TNF-α treatment

2 × 10^5^ LADC and LSCC cells were seeded in 12-well plates, and 24 h later they were treated with human recombinant TNF (10 ng/ml) for 2, 4, and 6 h.

### TNFR1 complex I immunoprecipitation

Cells were seeded in 15-cm dishes and treated as indicated with 3x FLAG-hTNF (5 mg/ml). To terminate stimulation, medium was removed, and plates were washed with 50 ml of ice-cold PBS. Plates were frozen at −80°C until all time points were acquired. Plates were thawed on ice, and cells were lysed in 1% Triton X-100 lysis buffer (30 mM Tris-HCl, pH 7.4, 120 mM NaCl, 2 mM EDTA, 2 mM KCl, 10% glycerol, and 1% Triton X-100, supplemented with protease inhibitors and PR619 [10 µM]). Cell lysates were rotated at 4°C for 20 min then clarified at 4°C at 14,000 rpm for 30 min. Proteins were immunoprecipitated from cleared protein lysates with 20 ml of anti-FLAG M2 beads (Sigma) with rotation overnight at 4°C. For the 0-h sample, 5 mg/ml of FLAG-TNF was added after lysis. Samples were washed four times in 1% Triton X-100 buffer with PR619 (10 µM) and eluted by boiling in 60 ml 1× SDS loading dye.

### Statistics

Statistical analysis was performed using GraphPad Prism software. Student’s *t* tests and one-way or two-way ANOVA were used to generate P values, as indicated in the figure legends. For Fig. 8 B, a log-rank (Mantel–Cox) test was used. A P value <0.05 was considered significant.

### Online supplemental material

Fig. S1 shows gene alteration data from TCGA, additional IHC of KF tumor samples, and IHC data from the Ad5-CK5-Cre model. Fig. S2 shows data from the CK19-Cre model. Fig. S3 shows IHC data from the lung tumor models in Fig. 2. Fig. S4 shows validation of LADC and LSCC cells isolated from CKFY mice, comparison of Fbw7 substrates in LADC and LSCC cells, and increase in LUBAC alterations and NF-κB target gene expression in human and murine LSCC, respectively. Fig. S5 shows the effect of combination treatment on human lung tumor cell lines, IHC quantifications from combination-treated animals, and toxicity assessments for the combination treatment. Tables S1 and S2 show primers and siRNA target sequences. Table S3 shows antibodies.
